# A Triphenylphosphonium-Functionalized Mitochondriotropic Nanocarrier for Efficient Co-Delivery of Doxorubicin and Chloroquine and Enhanced Antineoplastic Activity

**DOI:** 10.3390/ph10040091

**Published:** 2017-11-21

**Authors:** Katerina N. Panagiotaki, Zili Sideratou, Spiros A. Vlahopoulos, Maria Paravatou-Petsotas, Michael Zachariadis, Nikolas Khoury, Vassilis Zoumpourlis, Dimitris Tsiourvas

**Affiliations:** 1 Institute of Nanoscience and Nanotechnology, NCSR ‘‘Demokritos”, 15310 Aghia Paraskevi, Greece; knpanagiotaki@gmail.com (K.N.P.); z.sideratou@inn.demokritos.gr (Z.S.); 2 Ηoremeio Research Laboratory, First Department of Paediatrics, National and Kapodistrian University of Athens, 11527 Athens, Greece; sblachop@med.uoa.gr; 3 Institute of Nuclear and Radiological Sciences and Technology Energy and Safety, NCSR ‘‘Demokritos”, 15310 Aghia Paraskevi, Greece; mparavatou@rrp.demokritos.gr; 4 Institute of Biosciences and Applications, NCSR ‘‘Demokritos”, 15310 Aghia Paraskevi, Greece; mizachar@bio.demokritos.gr; 5 Institute of Biology, Medicinal Chemistry and Biotechnology, National Hellenic Research Foundation, 11635 Athens, Greece; nikos_kh1@hotmail.com (N.K.); vzub@eie.gr (V.Z.)

**Keywords:** drug delivery systems, mitochondrial targeting, drug combinations, poly(ethyleneimine), triphenylphosphonium cation, old drugs, chloroquine, doxorubicin

## Abstract

Drug delivery systems that target subcellular organelles and, in particular, mitochondria are considered to have great potential in treating disorders that are associated with mitochondrial dysfunction, including cancer or neurodegenerative diseases. To this end, a novel hyperbranched mitochondriotropic nanocarrier was developed for the efficient co-delivery of two different (both in chemical and pharmacological terms) bioactive compounds. The carrier is based on hyperbranched poly(ethyleneimine) functionalized with triphenylphosphonium groups that forms ~100 nm diameter nanoparticles in aqueous media and can encapsulate doxorubicin (DOX), a well-known anti-cancer drug, and chloroquine (CQ), a known chemosensitizer with arising potential in anticancer medication. The anticancer activity of this system against two aggressive DOX-resistant human prostate adenocarcinoma cell lines and in in vivo animal studies was assessed. The co-administration of encapsulated DOX and CQ leads to improved cell proliferation inhibition at extremely low DOX concentrations (0.25 μΜ). In vivo experiments against DU145 human prostate cancer cells grafted on immunodeficient mice resulted in tumor growth arrest during the three-week administration period and no pervasive side effects. The findings put forward the potential of such targeted low dose combination treatments as a therapeutic scheme with minimal adverse effects.

## 1. Introduction

One common theme in several types of antineoplastic drugs is their capacity to induce detectable levels of cell stress, which results in the stimulation of systems that degrade biomolecules [1]. Increased proteolysis of apoptosis mediators enables neoplastic cells to survive in spite of targeted biological therapy and treatment with established antineoplastic drugs [1,2]. It is often noted that cell lines established from cancer patients derive from tumors exposed to chemotherapy and show increased viability upon exposure to conditions that induce cell stress [1,3]. As a result, disruption of systems for material turnover can sensitize cancer cells to chemotherapy and biological therapy [1,4]. Analysis of intracellular signal transduction pathways operating in cancer leads to the conclusion that interactions between transcription factors, which mediate coordination between innate and adaptive immunity, are compromised in neoplastic tissue [5]. This aberrant function of transcription regulators occurs in parallel with changes in organelle homeostasis that encompass both cancer cells, as well as their microenvironment [1,5,6]. Hence, agents interfering with organelle homeostasis would be expected to affect tissue levels of cytokines, as well as subsets of malignant cells themselves. A drug that interferes potently with the response of cancer cells to cell stress is chloroquine.

Chloroquine (CQ), a drug used for decades against malaria, is increasingly assayed as a radiosensitizing and chemosensitizing substance for the treatment of cancer [7]. Until now, however, most clinical trials have failed to demonstrate a substantial antineoplastic effect for CQ [8,9]. Completed clinical trials have not given results that would allow CQ to become incorporated in everyday practice. One of several potential explanations is an insufficient penetration of CQ into tumor cells, due to the acidic microenvironment of the tumor tissue [10]. It has been recently shown that CQ entry into cancer cells can be inhibited in an acidic environment commonly encountered in densely-grown cancerous tissues [11,12]. The acidic microenvironment of tumor tissue plays an important role in the behavior of cancer cells and in their resistance to antineoplastic treatment [13]. Therefore, diverse analogues (hybrid molecules) of chloroquine are currently being developed [14]. While CQ has shown some potential to enhance antineoplastic effects of nanocarrier-based drugs [7,15], only recently have studies on nanocarriers co-encapsulating CQ, either as an autophagy inhibitor or to circumvent multidrug resistance, with known antineoplastic drugs, such as docetaxel or doxorubicin, been published [16,17].

A critical aspect for CQ impact is the targeted organelle: this would enable identification of modulators for selective antineoplastic activity. Targeting of organelles is very important for antineoplastic drugs as a means to enhance their efficacy [18]. The best example is the frontline drug doxorubicin (DOX), a DNA intercalator and damaging agent, that was one of the most effective therapies for prostate cancer [19], but due to its dose-limiting cardiotoxicity [20] and the chemo-resistance observed in prostate cancer, its use was diminished [21]. To overcome this drawback, several combination strategies have been proposed [22,23]. However, in vitro data show that mitochondria-targeted doxorubicin lacks any direct nuclear effects in cultured rat cardiomyocytes and that these cells are able to undergo mitochondrial biogenesis [20].

Recently, much attention has been directed towards targeting bioactive compounds to cell mitochondria, in order to remediate mitochondrial dysfunctions that are the cause of a number of diseases, including cancer [24,25]. One approach typically employed to target drugs to mitochondria is to conjugate the bioactive molecule of choice directly to mitochondriotropic ligands such as the triphenylphosphonium group (TPP) [26,27]. This, however, limits the application to one type of drug in each case and, furthermore, raises concerns on the biological activity of the chemically-conjugated drugs. Alternatively, mitochondriotropic drug delivery systems could be proven more efficient since they can encapsulate and transport a number of diverse bioactive molecules without chemically modifying them [25,26,27,28,29].

We previously demonstrated the capacity of a nanocarrier based on an oligolysine scaffold by addition of two triphenylphosphonium cations per oligomer to target mitochondria [30]. We were subsequently able to show efficient delivery of doxorubicin into mitochondria of cultured cells by triphenylphosphonium-functionalized poly(ethyleneimine) hyperbranched nanocarrier P-TPP-DOX, resulting in rapid cytotoxicity, mainly evident as necrosis [31]. Necrosis is known to increase by agents that interfere with acidification of mitochondria [32], and as CQ is a weak base, it could be expected to enhance necrosis, if targeted to mitochondria. The aim of the present study is to use this novel hyperbranched polymer-based mitochondriotropic nanocarrier for efficient co-delivery to cell mitochondria of doxorubicin, a known anticancer drug, and chloroquine, a drug that interferes potently with the response of cancer cells to cell stress, and further assay the activity of this system against adenocarcinoma cell lines. To this end, the introduction of the lipophilic decyltriphenylphosphonium (decylTPP) group to a hyperbranched poly(ethyleneimine) (PEI), having an average molecular weight of 1300, was accomplished through amide bond formation. The resulting decyltriphenylphosphonium-functionalized poly(ethyleneimine), P-TPP, being water insoluble, had the tendency to form colloidal particles in water. It was, however, possible to control the hydrophobic assembly of TPP-functionalized PEI molecules into ~100 nm mean diameter nanoparticles (P-TPP) and proceed with the encapsulation of DOX and CQ, affording P-TPP-DOX and P-TPP-CQ, respectively. The cellular uptake of DOX and the intracellular localization of DOX were confirmed by flow cytometry and confocal laser scanning microscopy (CLSM), respectively. Their impact on the viability of human prostate adenocarcinoma DU145 and PC3 cell lines was assessed. Finally, we assayed the antineoplastic activity of combined administration of P-TPP-DOX and P-TPP-CQ on prostate adenocarcinoma DU145 xenografts on SCID mice [33], in comparison to the administration of either free DOX or P-TPP-DOX.

## 2. Results

The conjugation of decyltriphenylphosphonium groups to PEI was achieved through the reaction of primary amines with the carboxylic group of (10-carboxydecyl)triphenylphosphonium bromide [30,31]. By precisely selecting the molar ratio of the interacting moieties and the synthetic conditions, it is possible to fine-tune the number of decylTPP groups attached in each poly(ethyleneimine) scaffold. As determined by IG ^13^C-NMR spectroscopy [31,34], by integrating the peaks at 36.6–39.6 ppm attributed to the α carbons of PEI relative to the newly-formed amide moieties (CH_2_NHCO), the α carbons relative to the remaining primary amino groups (NH_2_CH_2_) and the peak at 35.8 ppm attributed to the α carbons of the alkyl spacer relative to the newly-formed amide moieties (NHCOCH_2_), it was found that 2.8 ± 0.3 decylTPP groups were attached to each PEI scaffold. This number is optimum for achieving the hydrophilic/lipophilic balance needed to render the resulting compound water-insoluble, but able to produce nanoparticles in buffered media such as PBS or Opti-MEM. It should be noted that lower substitutions result in water-soluble materials, while higher ones lead to compounds that are completely water insoluble, forming coarse-grained particles on the level of micrometers in water.

Through the addition in Opti-MEM of 100 μL of a DMSO solution of P-TPP having a concentration of 90 μg/mL, aqueous dispersions of nanoparticles having a radius of 30–35 nm, as determined by DLS, can be prepared. Similar hydrodynamic radii were observed in the case of DOX-loaded P-TPP nanoparticles (Supplementary Materials, Figure S1). The amount of encapsulated DOX into P-TPP nanoparticles, determined after the removal of the non-encapsulated DOX by ultracentrifugation, was 50 μΜ in a dispersion containing 1 mg/mL P-TPP nanoparticles (loading capacity 3% *w/w*). CQ-loaded nanoparticles that were prepared in a similar manner, as detailed in the Experimental Section, had a hydrodynamic radius of 50–55 nm (Supplementary Materials, Figure S2) and a final concentration of 300 μΜ in a dispersion containing 1 mg/mL P-TPP nanoparticles (loading capacity 9%).

### 2.1. In Vitro Intracellular Uptake

**Figure 1:**
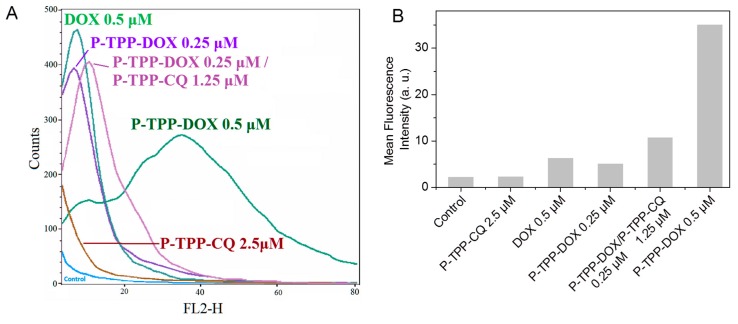
(**A**) Flow cytometry profiles of DOX fluorescence and (**B**) quantitative analysis of mean fluorescence intensity within DU145 human prostate cancer cells incubated with free DOX (0.5 μM), with P-TPP nanocarrier encapsulating DOX (P-TPP-DOX, DOX concentrations 0.25 μM and 0.5 μM) or chloroquine (P-TPP-CQ, CQ concentration 2.5 μM) and with both P-TPP-DOX and P-TPP-CQ (P-TPP-DOX 0.25 μM/P-TPP-CQ 1.25 μM).

The intracellular live-cell uptake of DOX was followed by fluorescence-activated cell sorting flow cytometry experiments since DOX can intercalate into mitochondrial DNA. The fluorescence emission profiles obtained from DU-145 cells treated with DOX or DOX encapsulated in P-TPP, with or without the co-addition of encapsulated CQ, are shown in Figure 1. It should be noted that in all cases, low DOX and CQ concentrations were used to avoid substantial cell death during the 2-h incubation period. Based on the obtained histograms, the addition of P-TPP-DOX at a DOX concentration of 0.25 μΜ exhibited a cellular uptake comparable to that of free DOX of twice the concentration (0.5 μΜ), while administration of P-TPP-DOX at 0.5 μΜ DOX concentration leads clearly to substantially higher cell uptake compared to free DOX also at 0.5 μΜ. The profile obtained following co-administration of P-TPP-CQ with P-TPP-DOX signifies that the presence of CQ encapsulated in P-TPP does not reduce P-TPP-DOX uptake due to possible interference, but instead appears to increase, to a small extent, DOX uptake. The respective controls (i.e., non-treated cells or cells treated with P-TPP-CQ) are also included for comparison purposes showing, as expected, minor self-fluorescence profiles of the cells. Consequently, intracellular uptake of DOX is enhanced when encapsulated in P-TPP, and the presence of P-TPP-CQ does not reduce this uptake.

### 2.2. Confocal Microscopy Studies

**Figure 2:**
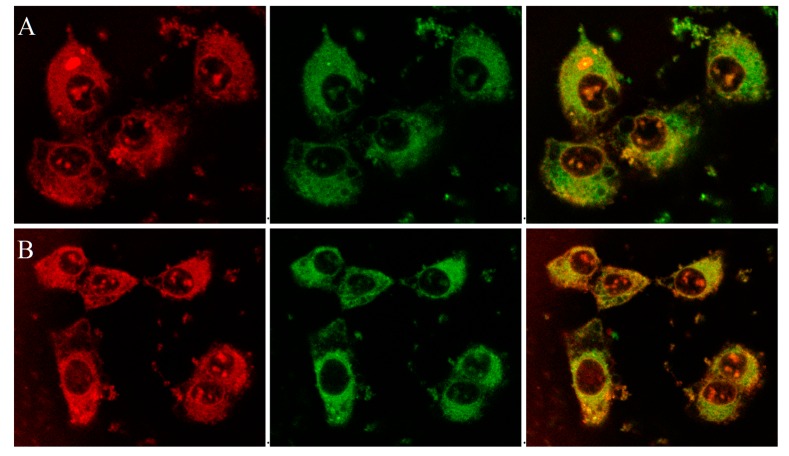
Confocal fluorescence microscopy on live DU145 cells incubated (**A**) with P-TPP-DOX (0.5 μΜ DOX concentration) or (**B**) with P-TPP-DOX/P-TPP-CQ (0.5 μΜ DOX, 1.25 μΜ CQ) for 1 h and 100 nM MitoTracker^®^ Green FM for 15 min: (left) DOX (red channel, λ_ex_ = 561 nm, λ_em_ = 570–650 nm); (middle) MitoTracker^®^ Green FM (green channel, λ_ex_ = 488 nm, λ_em_ = 500–550 nm); and (right) overlay image showing co-localization of green and red fluorescence in yellow.

The mitochondriotropic characteristic of the P-TPP nanocarrier, as previously established [31], has been utilized for targeting DOX in mitochondria by administering P-TPP-DOX to human prostate carcinoma DU145 cells. In this study, further laser scanning confocal microscopy (CLSM) studies have been employed on live DU145 cells, utilizing the intrinsic fluorescence of DOX and its ability to intercalate into DNA, in order to verify its intracellular localization after administering P-TPP-DOX either alone or together with P-TPP-CQ. It is well established that DOX encapsulated in P-TPP is localized in mitochondria [31] in contrast to free DOX, which is specifically localized in cell nuclei [31,35], and our confocal microscopy studies further corroborate this notion. Indeed, from the corresponding CLSM images (Supplementary Materials, Figure S3), DOX is predominantly located in the nucleus or its membrane, while no co-localization is observed with MitoTracker^®^ Green FM (Cell Signaling Technology, Inc., Beverly, MA, USA). On the contrary, as shown in the obtained confocal indicative images of cells treated with P-TPP-DOX at a 0.5 μΜ DOX concentration (Figure 2A and Figure S4A), it is evident that DOX is not located in the cell nuclei, but it is efficiently co-localized with MitoTracker^®^ Green FM in cell mitochondria (green + red = yellow). This result corroborates the already established mitochondrial affinity of P-TPP-DOX. At even lower DOX concentrations (0.25 μΜ DOX encapsulated in P-TPP), the corresponding CLSM images (Supplementary Materials, Figures S5 and S6) still show, although in less detail, that DOX is localized outside the cell nuclei. Live cell images obtained following co-administration of P-TPP-CQ with P-TPP-DOX (Figure 2B and Figure S4B) also exhibit the same distinct fluorescence pattern suggesting DOX mitochondrial localization even in the presence of P-TPP-CQ at various concentrations. The findings support the fluorescence emission profiles obtained from flow cytometry experiments and ascertain that administration of CQ encapsulated in P-TPP does not negatively compete with DOX internalization.

### 2.3. Cell Viability Studies

**Figure 3:**
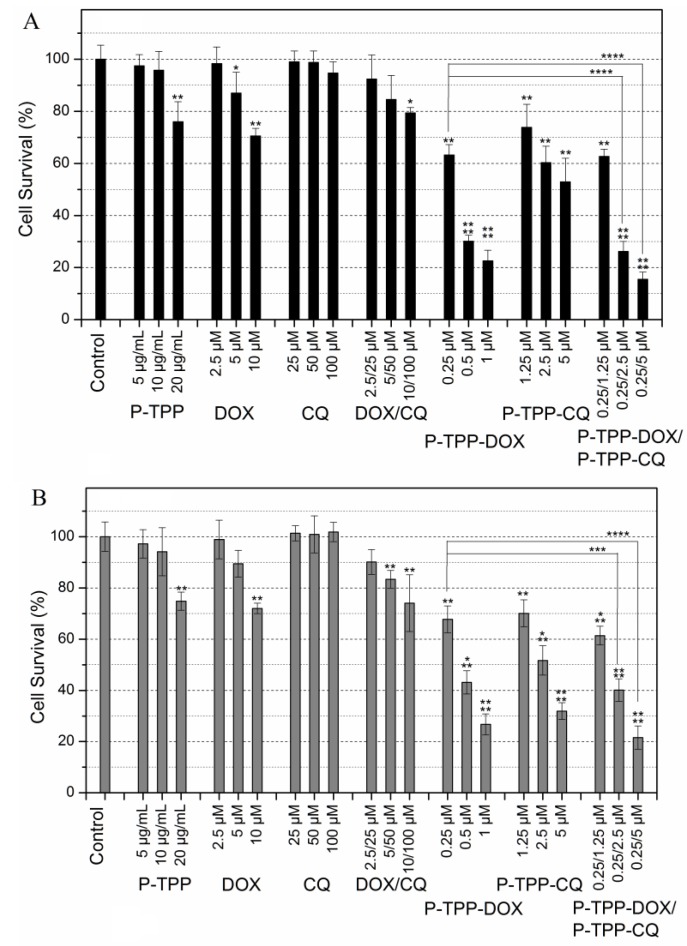
Comparative toxicities of P-TPP (carrier), free DOX, CQ and their combination, as well as of P-TPP-DOX, P-TPP-CQ or their combination on PC3 (**A**) and DU145 cells (**B**) following incubation at various concentrations for 3 h as determined by MTT assays 24 h following incubation. In all P-TPP-DOX experiments, the encapsulated DOX concentration was 0.25, 0.5 or 1 μM, and the corresponding carrier concentration was 5, 10 and 20 μg/mL (corresponding to 1.75, 3.5 and 7.0 μM of P-TPP). The statistical significance, resulting from Student’s paired two-tailed *t*-tests, follows the assignment: * *p* < 0.05, ** *p* < 0.01, *** *p* < 0.001 and **** *p* < 0.0001, while no annotation implies no statistical significance, *p* > 0.05.

Cell cytotoxicity studies were performed on PC3 and DU145 cell lines that are known to exhibit chemo-resistance against DOX and similar anti-cancer compounds [36,37,38,39]. Cell survival of both cell lines is shown in Figure 3. It becomes evident that under the experimental conditions employed (3-h incubation period, MTT assay 24 h following incubation), both cell lines exhibit noteworthy DOX resistance since a cell viability of ~70% is observed at the highest tested DOX concentrations of 10 μM. On the other hand CQ cytotoxicity is negligible even at high concentrations (100 μM) in line with the general knowledge that CQ is not cytotoxic vs. cancer cell lines [8,9]. Co-administration of free DOX and CQ results in a minor, non-statistically-significant, increase of cytotoxicity compared to that of free DOX. In line with our previously-published report [31], DOX encapsulated in P-TPP exhibits significant cytotoxicity at very low DOX concentrations with an IC_50_ below 0.5 μΜ against both cell lines. It is of interest to note that CQ encapsulated in P-TPP, in direct contrast to the non-encapsulated CQ, has a prominent effect on cell viability, as well, albeit at a much higher dose compared to DOX (IC_50_ ca 5 μΜ). Finally co-administration of P-TPP-DOX and P-TPP-CQ further reduces cell viability. Employing P-TPP-DOX at the lowest tested DOX concentration of 0.25 μM and P-TPP-CQ at various concentrations, the resulting effect on cell viability is further enhanced. This increase in cytotoxicity is statistically significant (*p* < 0.0001) when the CQ concentration is above 2 μM (Figure 3), signifying that by using an appropriate delivery system, severe cytotoxicity can be obtained at an extremely low DOX concentration (0.25 μM) by co-administering CQ also at a low concentration (2.5 μM). The toxicity of the P-TPP carrier was tested employing concentrations in the range of 5–20 μg/mL (equivalent to 1.75–7.0 μM of P-TPP) that correspond to the amounts of the carrier present in the above cell experiments employing P-TPP-DOX and P-TPP-CQ. It was found that the carrier exhibits some cytotoxicity (cell viability of ca. 80%) towards both cell lines, but only at high concentrations (20 μg/mL or 7.0 μM P-TPP). Cell viability studies were also performed on normal (non-cancerous) 3T3 mouse fibroblast cells under the same experimental conditions as above. The obtained results (Supplementary Material, Figure S7) show that the carrier and the free (non-encapsulated) CQ have the same negligible cytotoxicity as registered for PC3 and DU145 cell lines, while both free DOX and free DOX/CQ are considerably more cytotoxic for 3T3 than for PC3 and DU145. This is anticipated since the malignant cell lines are DOX resistant. On the other hand, P-TPP encapsulated DOX, CQ or DOX/CQ shows no statistically-significant different cytotoxicity profiles between the fibroblast cell line and the cancerous cell lines, suggesting that their formulations are equally cytotoxic for all three cell lines. Apparently, the carrier can equally target the mitochondria of both DOX-resistant or non-DOX-resistant cells.

### 2.4. In Vivo Animal Studies

**Figure 4:**
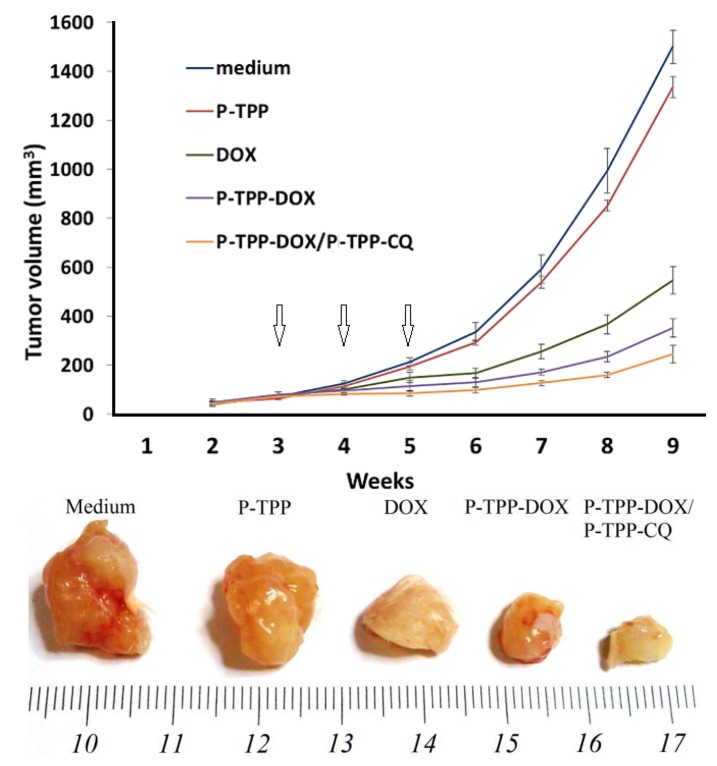
CQ and DOX encapsulation in P-TPP reduces DU145 tumor growth in SCID mice in comparison to treatment with free or nanocarrier-encapsulated DOX. (**Upper part**): Growth rates of tumor xenografts in SCID mice. Formulations were administered intraperitoneally on Weeks 3, 4 and 5 (arrows). Group 1 mice were treated with medium (Opti-MEM); Group 2 with nanocarrier P-TPP (75 mg/kg); Group 3 with DOX (2 mg DOX/kg); Group 4 with P-TPP-DOX (0.75 mg/kg); Group 5 with P-TPP-DOX (0.75 mg/kg)/P-TPP-CQ (4.5 mg/kg). (**Lower part**): Representative images of tumors excised after nine weeks (experimental endpoints).

To investigate with in vivo studies the activity of P-TPP encapsulated DOX and CQ, we grafted the cell line DU145 into SCID mice, since DU145 cells are refractory to androgens and do not express the ARα or ERα [40]. To this end, we administered free DOX, P-TPP encapsulated DOX and the combination of P-TPP-DOX with P-TPP-CQ once a week and for a total period of three weeks and compared the results with the control group (medium only) and the group administered the nanocarrier at an equivalent concentration. Co-administration of P-TPP encapsulated CQ and DOX had the greatest impact on tumor growth, being significantly more potent than DOX (*p* < 5.1 × 10^−6^) and P-TPP-DOX (*p* < 0.001), as well (Figure 4). Specifically, tumors in the control and in vehicle-treated animals reached exponential growth kinetics (Figure 4, upper diagram). In comparison, tumor growth in DOX-treated animals was attenuated, and the P-TPP-DOX- and P-TPP-DOX/P-TPP-CQ-treated animals manifested the most significant growth attenuation. Especially during the three-week administration period (marked with arrows in Figure 4), complete tumor growth arrest was observed for mice administered with P-TPP-DOX/P-TPP-CQ. In addition, both animal groups treated with P-TPP-DOX and P-TPP-DOX/P-TPP-CQ exhibited normal movement capability, and normal, expected overall behavior. Finally, no pervasive side effects could be noted during the observation period for these groups. 

## 3. Discussion

In this study, first, it is noted that both free and encapsulated DOX kill significant scores of DU145 and PC3 cells both in vitro and in vivo. It is also clear that a synergistic cytotoxicity on both cell lines upon co-treatment with CQ and DOX, both encapsulated in the triphenylphosphonium-functionalized nanocarrier, was observed, and this cytotoxic impact is substantially increased upon addition of increasing quantities of encapsulated CQ. This increase of cytotoxicity has an added value, because the two drugs act with complementary mechanisms to yield oncosuppressive effects. 

Currently, CQ is mainly assayed as a substance that could sensitize some types of tumor cells to antineoplastic treatment [10,12]. However, its effects vary depending on complex aspects of the cancer phenotype, which relate to the metabolic state of multiple cohorts of cells within the cancer microenvironment [1,5]. The mechanism whereby this carrier exerts its impact on these cell lines, leading to the principal difference between free CQ and P-TPP-CQ, is possibly related to changes in the capacity of CQ to enter the cells. Even though CQ has complex effects on cells [7,12], its main target that relates to cellular turnover is the lysosome, and consequentially, CQ inhibits the formation of the autophagosome in cells undergoing autophagy. Thereby, CQ inhibits late stage autophagy: a recent study with isoquinoline alkaloid liensinine has suggested that breast cancer cells can be killed by inhibiting late stage autophagy and specifically the fusion of autophagosome with lysosome; and not by blocking the early stage, i.e., the formation of autophagosome [41]. In previous studies, breast cancer cells resistant to chemotherapy were killed by inducing endoplasmic reticulum (En.R) stress [42]. The combination of En.R stress induction with CQ has indicated a potential for synergistic cytotoxicity. As an example, CQ has shown a remarkable potential to shrink xenografts of triple-negative breast cancer cells upon combination treatment aiming to cause endoplasmic reticulum stress [43]. 

In a previously-published article, we demonstrated that P-TPP encapsulated doxorubicin targeted mitochondria [31]. Similar studies have shown that TPP-conjugated dendrimers enable targeting mitochondria as gene transfection vectors [44,45] and, more generally, that TPP functionalized nanoparticles also exhibit mitochondriotropic properties [26,27,46]. In the present study, a cooperative effect was observed upon co-administration of DOX and CQ that are both encapsulated in P-TPP. By targeting mitochondria, P-TPP could be anticipated to modulate the effects of CQ on cells: recently, scanning electron microscopy and tomography were used to identify membrane contact sites between cellular organelles and the En.R concluding that multiple organelles, including lysosomes and late endosomes, form contact sites with mitochondria and En.R [47]. Therefore, tumor cells that degrade tumor suppressor proteins at the membranes forming the interface between mitochondria and En.R [48] could be eradicated by agents that reach this interface and simultaneously block organelle acidification. 

Indeed, doxorubicin resistance is associated with delivery of damaged mitochondria to autophagosomes, a process that is inhibited by CQ [49]. As doxorubicin mainly damages DNA, it is relevant to emphasize that CQ, acting as an autophagy inhibitor, interferes with the repair of damaged DNA and induces mitochondrial cristae damage. This leads to mitochondrial membrane depolarization with a significant reduction in the activity of cytochrome c oxidase and accumulation of superoxide and double-stranded DNA breaks [50,51]. As a result, CQ and DOX co-administration shows a substantial oncosuppressive activity on aggressive tumor xenografts (Figure 4). In vitro, however, the synergy of TPP-encapsulated CQ and DOX was modest (Figure 3). In the mammalian organism, CQ has multiple targets that influence diverse aspects of tissue physiology and immunity. CQ has the potential to affect cancer cell invasion, chemotaxis, transdifferentiation and clonogenicity, through several mechanisms [12]. In mice, CQ shows a significant potential to decrease inflammatory macrophage activity and oxidant stress, normalizing the signaling pathways that regulate the function of cells of the monocyte/macrophage lineage and permitting the activity of cytotoxic T-cells [52,53,54]. CQ prolonged the survival of mice bearing breast cancer xenografts, showing an impact on both macrophages and T-cells [54]. The cooperation of macrophages and T-cells is critical in suppression of carcinogenesis [55]. Nevertheless, the presence of basic elements of innate immunity in the SCID mouse can explain at least part of the in vivo CQ-DOX synergy. Natural killer cells can mediate potent oncosuppression through tumor necrosis factor-related apoptosis-inducing ligand (TRAIL) [56,57].

In SCID mice bearing tumor xenografts, DOX could augment oncosuppression by NK cells, an effect that in vitro was reproduced with DU145 cells. This was shown by using the SCID/beige mice that are NK cell-deficient, which then received either NK cells, or T-cells, or both, to ascertain the role of each cell type separately [56]. It is interesting to note that DU145 cells evade TRAIL-induced cell death by inducing autophagic flux, which is inhibited by CQ [58]. CQ therefore has the capacity to potentiate the DOX-induced oncosuppression by NK cells. Therefore, at least one potential explanation for the synergy of encapsulated DOX and CQ in vivo could be the enhancement of innate immunity against the SCID-mouse xenografted DU145 tumors. This aspect of drug synergy can have a significant impact on translational models of adoptive therapy.

## 4. Materials and Methods 

### 4.1. Chemicals and Reagents.

Opti-MEM without phenol red (Gibco) was purchased from ThermoFisher Scientific (San Jose, CA, USA). DMEM low glucose, with phenol red, fetal bovine serum (FBS), penicillin/streptomycin, l-glutamine, phosphate buffer saline (PBS) and trypsin/versene were all purchased from Biochrom (Berlin, Germany). Thiazolyl blue tetrazolium bromide (MTT), dimethyl sulfoxide (DMSO) and isopropanol were purchased from Merck KGaA (Calbiochem^®^, Darmstadt, Germany). *N*-hydroxybenzotriazole (HOBt) and 2-(1H-benzotriazole-1-yl)-1,1,3,3-tetramethyluronium (HBTU) were purchased from Anaspec (San Jose, CA USA). *N*,*N*-diisopropylethylamine (DIPEA) and chloroquine diphosphate were purchased from Sigma-Aldrich Ltd. (Poole, UK). MitoTracker^®^ Green (Cell Signaling Techology Inc., Beverly, MA, USA) FM was purchased from Cell Signaling Technology, Inc. (Beverly, MA, USA). Doxorubicin hydrochloride and hyperbranched poly(ethyleneimine) (PEI) of 1300 molecular weight (Lupasol^®^ G20, water-free, 98%) were kindly donated by Regulon SA (Athens, Greece) and BASF (Ludwigshafen, Germany), respectively.

### 4.2. Synthesis of P-TPP

**Scheme 1:**
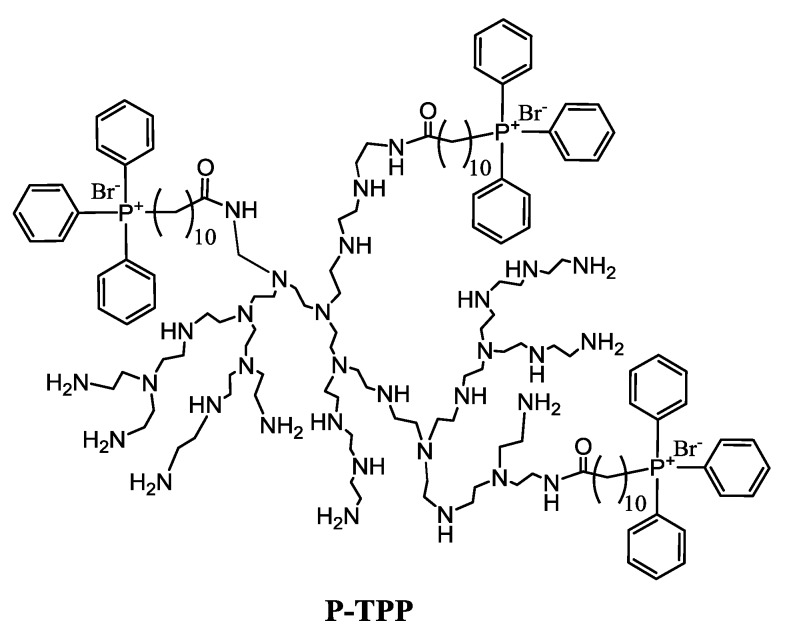
Schematic representation of P-TPP.

The introduction of decyltriphenylphosphonium (decylTPP) groups to PEI, having on average 10 primary amine groups as determined by inverse-gate decoupling ^13^C-NMR, was realized in two steps as detailed in a previous publication [31]. In brief, in the first step, (10-carboxydecyl)triphenylphosphonium bromide was obtained by reacting triphenylphosphine with 11-bromoundecanoic acid in anhydrous acetonitrile at a 1:1.1 molar ratio [30]. Subsequently, in the second step, (10-carboxydecyl)triphenylphosphonium bromide dissolved in anhydrous DMF was allowed to react overnight, under inert atmosphere, with the primary amino groups of PEI at a 1:4 molar ratio, in the presence of HBTU, HOBt (1.6 mmol) and DIPEA (1.6 mmol). The product was twice precipitated with diethyl ether, and the structure of P-TPP was established by ^1^H- and ^13^C-NMR spectroscopy (Supplementary Materials, Figures S8 and S9). It was found that, on average, 2.8 ± 0.3 decylTPP groups were conjugated to PEI (schematically depicted in Scheme 1 with 3 decylTPP groups) as determined by inverse gated ^13^C-NMR, which corresponds to a mean molecular weight of 2830.

^1^H-NMR (500 MHz, MeOD-*d*_4_): δ = 1.10–1.40 (m, aliphatic CH_2_), 1.45–1.75 (m, CH_2_CH_2_P^+^Ph_3_, NHCOCH_2_CH_2_), 2.05–2.25 (t, NHCOCH_2_), 2.40–3.05 (m, CH_2_ of PEI skeleton, CH_2_NHCO), 3.35–3.55 (m, CH_2_P^+^Ph_3_), 7.55–7.90 (m, aromatic H).

^13^C-NMR (125.1 MHz, MeOD-*d*_4_): δ = 175.15 (C=O), 134.85 (P^+^Ph_3_ para), 133.50 (d, *J =* 11.5 Hz, P^+^Ph_3_ ortho/meta), 130.25 (d, *J* = 14.57 Hz, P^+^Ph_3_ ortho/meta), 124.15 (d, *J* = 13.56 Hz, P^+^Ph_3_ ipso), 53.00–49.30 and 46.40–43.90 (NCH_2_CH_2_N), 39.60–36.60 (NH_2_CH_2_, CH_2_NHCO), 35.80 (CONHCH_2_), 30.30 (d, *J* = 18.23 Hz, CH_2_CH_2_CH_2_P^+^Ph_3_), 29.50–28.10 (aliphatic CH_2_), 22.20 (CH_2_P^+^Ph_3_), 21.50 (d, *J* = 31.50 Hz, CH_2_CH_2_P^+^Ph_3_).

### 4.3. Preparation and Characterization of P-TPP Nanoparticles and of DOX- or CQ-Loaded Nanoparticles

P-TPP nanoparticles were prepared by the drop-wise addition in Opti-MEM (cell growth medium free of serum or phenol red) of a DMSO solution of P-TPP. These nanoparticles are able to encapsulate in their interior DOX [31], due to the intrinsic property of the hyperbranched polymeric architecture to form nanocavities in their interior, the environment of which determines their solubilizing, or encapsulating, properties towards a variety of labile or poorly-soluble drugs [59,60]. Drug encapsulation within the dendrimeric interior typically involves non-covalent bonding interactions between specific groups of the dendrimer and the drug. In this particular case, it is envisaged that DOX is residing in the interior of PEI due to hydrophobic interactions and/or charge-transfer interactions between the lone electron pair of tertiary nitrogens of PEI and the aromatic part of DOX [61,62].

For the preparation of drug-loaded P-TPP nanoparticles, the same procedure was followed with the drugs dissolved either in the DMSO solution (in the case of CQ) or in Opti-MEM (in the case of DOX). In a typical experiment, 100 μL of a 90 mg/mL P-TPP solution in DMSO were added drop-wise under vigorous stirring to 1 mL of Opti-MEM affording spontaneously-formed nanoparticles of ca. 100 nm hydrodynamic radii. For the preparation of DOX-loaded nanoparticles, 100 μL of a 90-mg/mL P-TPP solution in DMSO were added to a 1-mg/mL DOX solution in Opti-MEM. For the preparation of CQ-loaded nanoparticles, 100 μL of a 50 mg/mL CQ solution in DMSO also containing 90 mg/mL P-TPP were added as above to 1 mL of Opti-MEM. In order to remove non-encapsulated CQ or DOX, the obtained nanoparticles were centrifuged at 90,000 rpm for 45 min in an Optima^TM^ Max Ultracentrifuge Beckman Coulter, Inc. (Fullerton, CA, USA) coupled with the MLA-130 rotor. The obtained pellets were washed with Opti-MEM and centrifuged 3 times, to ensure complete removal of non-encapsulated CQ or DOX. The pellets were finally resuspended in 1 mL of Opti-MEM, and the suspensions were sonicated for 1 min using a standard sonotrode (3 mm tip-diameter) in a Hielscher UP200S high intensity ultrasonic processor (Hielscher-Ultrasound Technology, Teltow, Germany) at 50% amplitude and 0.5 cycles to finely disperse the nanoparticles. The resulting dispersions were syringe-filtered through sterile nylon membrane filters of a 0.20-μm pore size.

The size distributions of P-TPP and DOX- or CQ-loaded P-TPP nanoparticles were determined by dynamic light scattering (DLS) employing an AXIOS-150/EX (Triton Hellas) apparatus with a 30-mW laser source and an Avalanche photodiode detector at an angle of 90°. For these experiments, 200-μL dispersions of P-TPP or drug-loaded P-TPP nanoparticles were used. For each dispersion, at least ten light scattering measurements were collected, and the results were averaged.

For the determination of DOX encapsulated in P-TPP nanoparticles, for every batch prepared, a known amount of P-TPP-DOX nanoparticle dispersion (typically 100 μL) was dissolved in 1000 μL ethanol, and its absorbance at 500 nm was registered using a Cary 100 Conc UV-Visible spectrophotometer (Varian Inc., Mulgrave, Australia). The spectra of free DOX, P-TPP and P-TPP-loaded DOX are shown in the Supplementary Materials, Figure S10. Subsequently, DOX concentration was determined employing a calibration curve separately constructed from DOX standards (10–100 μM). Similarly, the CQ concentration was spectrofluorometrically determined by dissolving a known amount of P-TPP-CQ nanoparticles dispersion (typically 10 μL) in 1000 μL ethanol, registering the fluorescence intensity at 375 nm (excitation: 320 nm) in a Cary Eclipse fluorometer (Varian Inc.) and calculating the concentration employing a calibration curve constructed from CQ standards (2–15 μM). The fluorescence spectra of free and P-TPP-loaded CQ are shown in the Supplementary Materials, Figure S11.

### 4.4. Cell Culture

Cells used in this study were the human prostate carcinoma cell lines DU145, PC3 and 3T3 mouse fibroblasts obtained from the American Type Culture Collection (ATCC, Manassas, VA, USA). The cells were grown in DMEM with phenol red supplemented with 10% FBS, 1% penicillin/streptomycin and 1% l-glutamine at 37 °C in a 5% CO_2_ humidified atmosphere.

### 4.5. Confocal Microscopy

DU145 cells (10 × 10^5^) were inoculated on Nunc™ glass base dishes (ThermoFisher Scientific, Rochester, NY, USA) and left to grow overnight in 2 mL of DMEM complete media in the same conditions as elaborated earlier in the cell culture section. Cells were subsequently incubated with DOX, P-TPP-DOX and a combination of P-TPP-DOX with P-TPP-CQ for 1 h or 2 h in Opti-MEM. Specimens were examined on a multiphoton confocal microscope Leica TCS SP8 MP (Wetzlar, Germany) equipped with an Argon laser (excitation lines at 458, 476, 488, 496 and 514 nm), a DPSS 561 laser (excitation line at 561 nm) and an IR MaiTai DeepSee Ti:Sapphire laser (Spectra-Physics, Santa Clara, CA, USA) for multiphoton applications. Images were acquired with the spectral detector of the microscope using appropriate emission wavelength ranges; DOX (red) was excited at 561 nm with the DPSS laser, and emission was recorded between 570 and 650 nm, while MitoTracker^®^ Green FM was excited at 488 nm with the argon laser, and emission was recorded between 500 and 550 nm. Images were acquired with the LAS X software (Leica Microsystems CMS GmbH, Wetzlar, Germany) and are presented without any post-processing.

### 4.6. Flow Cytometry

The in vitro cellular uptake of free DOX and nanocarrier-encapsulated DOX or CQ was determined by flow cytometry. DU145 were grown overnight at a density of 25 × 10^4^ cells per well in 6-well plates. Subsequently, cells were exposed to free DOX, CQ or nanoparticle dispersions of P-TPP, P-TPP-DOX, P-TPP-CQ and their combination P-TPP-DOX/P-TPP-CQ for 2 h. The wells were then washed twice with phosphate-buffered saline and trypsinized, and the resulting cell suspensions were centrifuged for 5 min at 1000 rpm. Finally, the collected cells were resuspended in PBS, and the uptake of DOX was analyzed using a FACSCalibur flow cytometer (Becton Dickinson, Heidelberg, Germany).

### 4.7. MTT Viability Assay

DU145 or PC3 human prostate cancer cells were inoculated (10 × 10^3^) into 96-well plates and left to incubate in 100 μL DMEM containing 10% FBS, 1% penicillin/streptomycin and 1% l-glutamine for 24 h at 37 °C in a 5% CO_2_ humidified atmosphere. Cells were then treated with 100 μL nanoparticle dispersions of P-TPP, P-TPP-DOX, P-TPP-CQ and their combination P-TPP-DOX/P-TPP-CQ, at various concentrations, for 3 h in Opti-MEM. After this period, the nanoparticle-containing media were replaced with complete media as above and further incubated for 24 h. For comparison, cells were also treated with free (non-encapsulated) DOX or CQ solutions of corresponding concentrations as well as of DOX/CQ combinations. The mitochondrial redox function, translated as cell viability, was assessed by the MTT assay 24 h later. Cell medium in each well was replaced with 100 μL MTT solution (1 mg/mL in DMEM), and cells were incubated at 37 °C in a 5% CO_2_ humidified atmosphere for 4 h. MTT media were then removed from all cells, and the produced formazan crystals were solubilized with 100 μL 2-isopropyl ethanol per well. The plates were subsequently shaken for 10 min at 100 rpm in a Stuart SI500 orbital shaker, and the absorbance at 540 nm was measured using an Infinite M200 microplate reader (Tecan group Ltd., Männedorf, Switzerland). Blank values measured in wells with 2-isopropyl ethanol without cells were in all cases subtracted. The relative cell viability was determined as cell survival percentage compared to cells that were treated with complete media, which were used as the control.

### 4.8. Statistical Analysis

All experiments were repeated independently at least three times. MTT data are shown as means of six independent values with error bars representing one standard deviation. Student’s paired two-tailed *t*-test was performed on the MTT cytotoxicity data obtained to determine the statistical significance of a difference between means. Τhe statistical significance follows the assignment: * *p* < 0.05, ** *p* < 0.01, *** *p* < 0.001 and **** *p* < 0.0001; while no annotation implies no statistical significance: *p* > 0.05.

### 4.9. Tumor Xenografts

DU145 cells (1 × 10^6^) were resuspended in 100 μL PBS and injected subcutaneously into the left and right flanks of 6-week old female SCID mice (The Jackson Laboratory, Bar Harbor, ME, USA). This strain shows a severe combined immunodeficiency affecting both B and T lymphocytes; however, the mouse strain has normal natural killer (NK) cells (phenotype: severe combined immunodeficiency, autosomal recessive, T-cell-negative, B-cell-negative, NK cell-positive) [63]. SCID mice are routinely used in oncogenicity protocols to avoid bias due to immunological responses of mice to injected cells [33]. Mice were divided into five (5) groups of three. Tumors were allowed to grow for 2 weeks. At the end of the third week, each group of mice was treated once a week for a period of three weeks, with an intraperitoneal injection. Specifically, mice of Group 1 were treated with an injection of 300 μL Opti-MEM medium each; those of Group 2 were treated with 300 μL of the nanocarrier P-TPP corresponding to 75 mg/kg; Group 3 with 300 μL of DOX corresponding to a dose of 2 mg DOX/kg; Group 4 with 300 μL P-TPP-DOX corresponding to a dose of 0.75 mg/kg with respect to DOX and to 25 mg/kg with respect to P-TPP; while Group 5 received in total 300 μL of P-TPP-DOX/P-TPP-CQ, corresponding to a dose of 0.75 mg/kg with respect to DOX, 4.5 mg/kg with respect to chloroquine and to 75 mg/kg with respect to the nanocarrier P-TPP. During this period, tumor volumes were measured using a caliper and calculated using the formula ½ × height × width × length. The observation period lasted until tumors in control mice reached a volume of 1500 mm^3^. At the end of the observation period, mice were sacrificed, and tumors were excised and photographed.

### 4.10. Ethics Statement

All experiments with mice were performed in the authorized animal house of the National Hellenic Research Foundation. Experiments complied with the Protocol on the Protection and Welfare of Animals, as obliged by the rules of the National Hellenic Research Foundation, the regulations of the National Bioethics Committee and Article 3 of Presidential Decree 160/1991 (in line with the 86/609/EEC directive) regarding the protection of experimental animals.

## 5. Conclusions

Triphenylphosphonium-functionalized poly(ethyleneimine) nanoparticles can efficiently target mitochondria and can effectively encapsulate two different bioactive compounds. The administration of encapsulated DOX and/or CQ against two aggressive DOX-resistant human prostate adenocarcinoma cell lines DU145 and PC3 results in significantly reduced cell viability: although free CQ did not cause inhibition even at a dose of 100 μM, it was found that P-TPP-CQ strongly inhibited cell proliferation (IC_50_ 2.5–5.0 μΜ, depending on the cell line), while administration of DOX (up to 10 μΜ) caused minor cell death in direct contrast to P-TPP-DOX (IC_50_ 0.5 μΜ). Co-administration of P-TPP-DOX (0.25 μΜ) with P-TPP-CQ (2.5 μΜ) further enhanced cytotoxicity, indicating that this administration scheme is potent and could significantly reduce DOX side effects. Indeed, in vivo experiments with immunodeficient mice designate no pervasive side effects and tumor growth arrest during the three-week administration period. The observed synergy can be rationalized by the fact that chloroquine, a known autophagy inhibitor, can be effective in restoring doxorubicin action in doxorubicin-resistant tumors, which, however, is not sufficiently attained when non-encapsulated drugs were co-administered. Overall, the developed triphenylphosphonium-functionalized poly(ethyleneimine) nanoparticles can encapsulate DOX and CQ, efficiently target mitochondria and enhance the antineoplastic activity of both medications.

## Supplementary Materials

The following are available online at www.mdpi.com/1424-8247/10/4/91/s1: Figure S1: Intensity-weighted hydrodynamic radii size distribution of DOX-loaded P-TPP nanoparticles; Figure S2: Intensity-weighted hydrodynamic radii size distribution of CQ-loaded P-TPP nanoparticles; Figure S3: Confocal fluorescence microscopy on live DU145 cells incubated with 5 μΜ DOX for 2 h and 100 nM MitoTracker^®^ Green FM for 15 min; Figure S4: Confocal fluorescence microscopy on live DU145 cells incubated with P-TPP-DOX (0.5 μΜ DOX concentration) or with P-TPP-DOX/P-TPP-CQ (0.5 μΜ DOX, 1.25 μΜ CQ) for 2 h; Figure S5: Confocal fluorescence microscopy on live DU145 cells incubated with P-TPP-DOX/P-TPP-CQ (0.25 μΜ DOX, 1.25 μΜ CQ) for 2 h and 100 nM MitoTracker^®^ Green FM for 15 min and the overlay image; Figure S6: Confocal fluorescence microscopy on DU145 cells incubated for 2 h with PEI-TPP-DOX at a 0.25 μΜ DOX concentration; Figure S7: Comparative toxicities of P-TPP, free DOX, CQ and their combination, as well as of P-TPP-DOX, P-TPP-CQ or their combination on 3T3 cells following incubation at various concentrations for 3 h as determined by MTT assays 24 h following incubation; Figure S8: ^1^H-NMR (500 MHz, MeOD-*d*_4_) spectrum of P-TPP; Figure S9: ^13^C-NMR (125.1 MHz, MeOD-*d*_4_) spectrum of P-TPP; Figure S10: UV-Vis spectra of the carrier P-TPP, free DOX and P-TPP-DOX (DOX concentration 9.3 μM) in ethanol; Figure S11: Fluorescence spectra of CQ or P-TPP-CQ (CQ concentration 3.5 μM) in ethanol.
