# Supplementary material for: A Triphenylphosphonium-Functionalized Mitochondriotropic Nanocarrier for Efficient Co-Delivery of Doxorubicin and Chloroquine and Enhanced Antineoplastic Activity
Source: Pharmaceuticals (Basel). 2017 Nov 21;10(4):91. doi: 10.3390/ph10040091 (PMC5748647; doi:10.3390/ph10040091)
Supplement: Supplementary file 1 [file pharmaceuticals-10-00091-s001.pdf]

Supplementary Materials:
A Triphenylphosphonium-Functionalized Mitochondriotropic Nanocarrier for Efficient Co-Delivery of Doxorubicin and Chloroquine and Enhanced Antineoplastic Activity

Katerina N. Panagiotaki, Zili Sideratou, Spiros Vlahopoulos, Maria Paravatou-Petsotas, Michael Zachariadis, Nikolas Khoury, Vassilis Zoumpourlis, Dimitris Tsiourvas

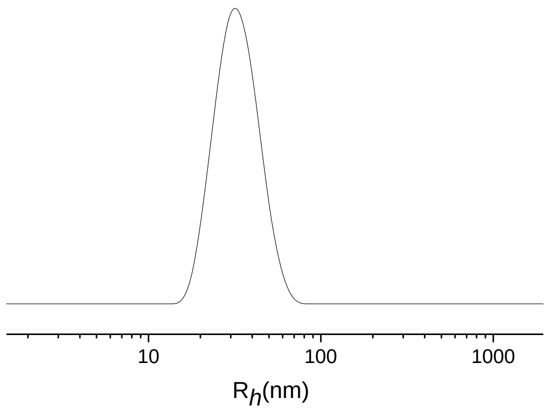

Figure S1. Intensity-weighted hydrodynamic radii size distribution of DOX-loaded P-TPP nanoparticles.

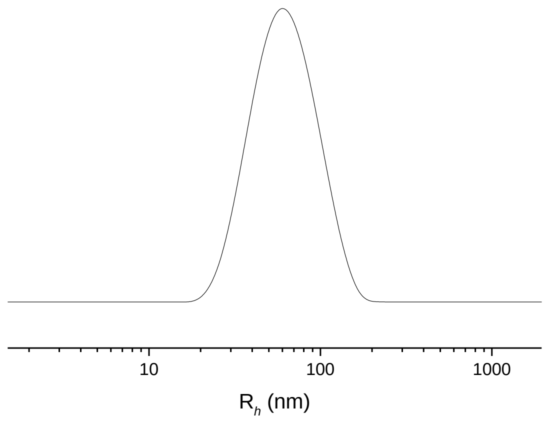

Figure S2. Intensity-weighted hydrodynamic radii size distribution of CQ-loaded P-TPP nanoparticles.

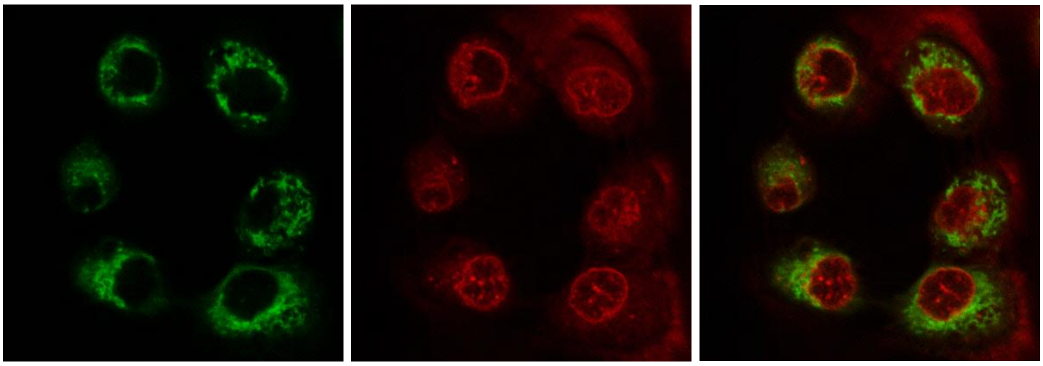

Figure S3. Confocal fluorescence microscopy on live DU145 cells incubated with DOX (5 μM DOX concentration) for 2 h and 100 nM MitoTracker® Green FM for 15 min: (left) DOX (red channel, λ_{ex} = 561 nm, λ_{em} = 570–650 nm); (middle) MitoTracker® Green (green channel, λ_{ex} = 488 nm, λ_{em} = 500–550 nm); and (right) overlay image showing co-localization of green and red fluorescence in yellow.

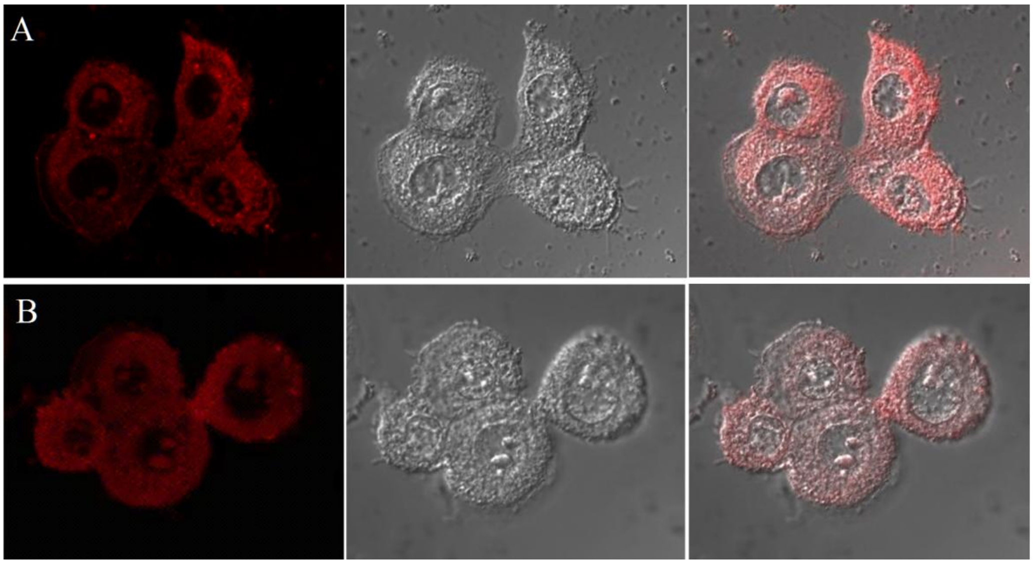

Figure S4. Confocal fluorescence microscopy on live DU145 cells incubated (**A**) with P-TPP-DOX (0.5 μM DOX concentration) or (**B**) with P-TPP-DOX/P-TPP-CQ (0.5 μM DOX, 1.25 μM CQ) for 2 h. The confocal fluorescence images, the corresponding brightfield images and their overlay are shown in the first, second and third column, respectively.

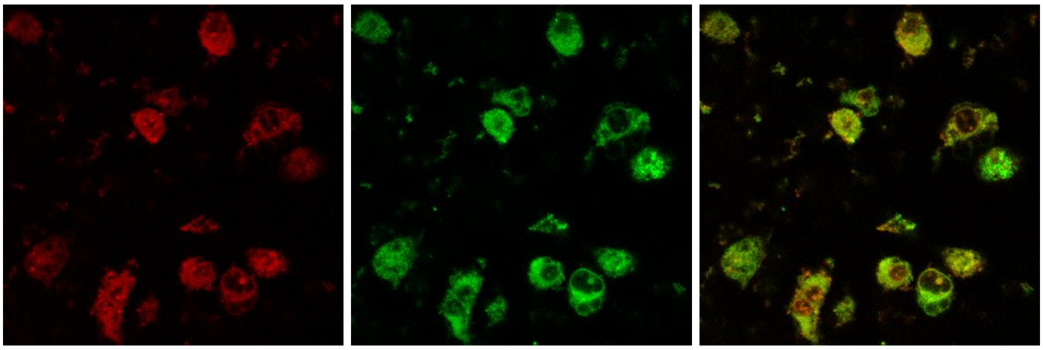

Figure S5. Confocal fluorescence microscopy on live DU145 cells incubated with P-TPP-DOX/P-TPP-CQ (0.25 μM DOX, 1.25 μM CQ) for 2 h and 100 nM MitoTracker® Green FM for 15 min: (left) DOX (red channel, λ_{ex} = 561 nm, λ_{em} = 570–650 nm); (middle) MitoTracker® Green (green channel, λ_{ex} = 488 nm, λ_{em} = 500–550 nm); and (right) overlay image showing co-localization of green and red fluorescence in yellow.

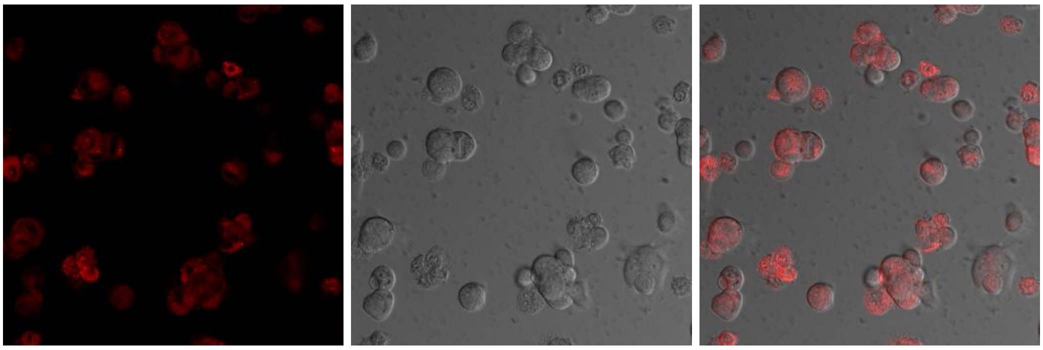

Figure S6. Confocal fluorescence microscopy on DU145 cells incubated for 2 h with poly(ethyleneimine) (PEI)-TPP-DOX at a 0.25 μM DOX concentration. The confocal fluorescence images, the corresponding brightfield images and their overlay are shown in the first, second and third column, respectively.

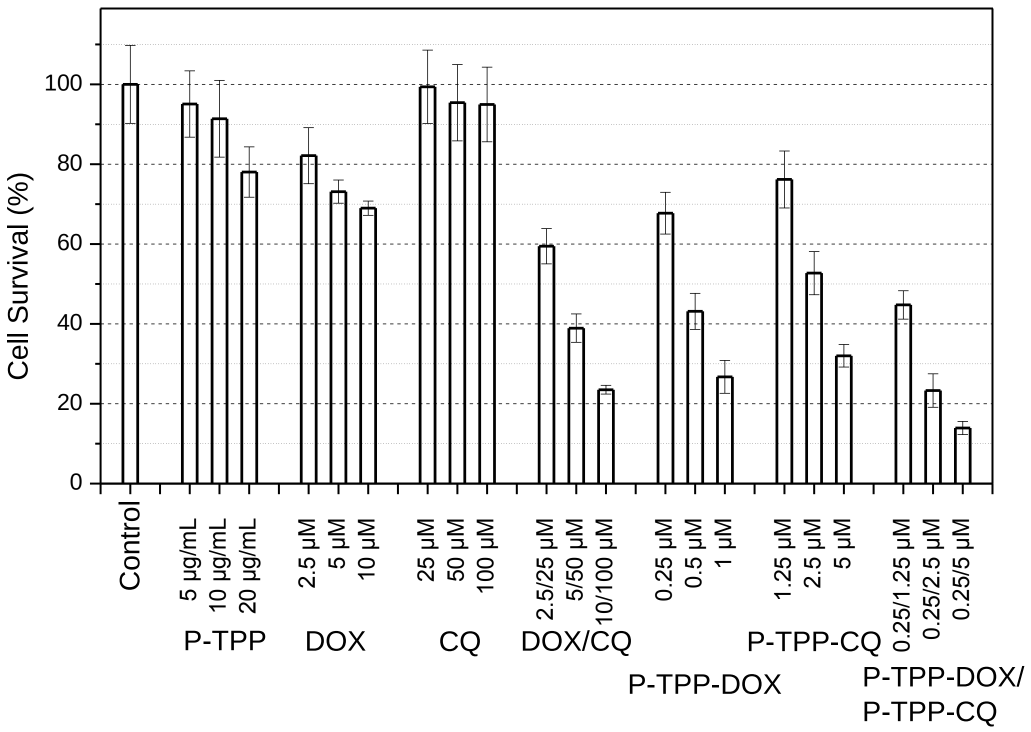

Figure S7. Comparative toxicities of P-TPP (vehicle), free DOX, CQ and their combination, as well as of P-TPP-DOX, P-TPP-CQ and their combination on 3T3 cells following incubation at various concentrations for 3 h as determined by MTT assays 24 h following incubation.

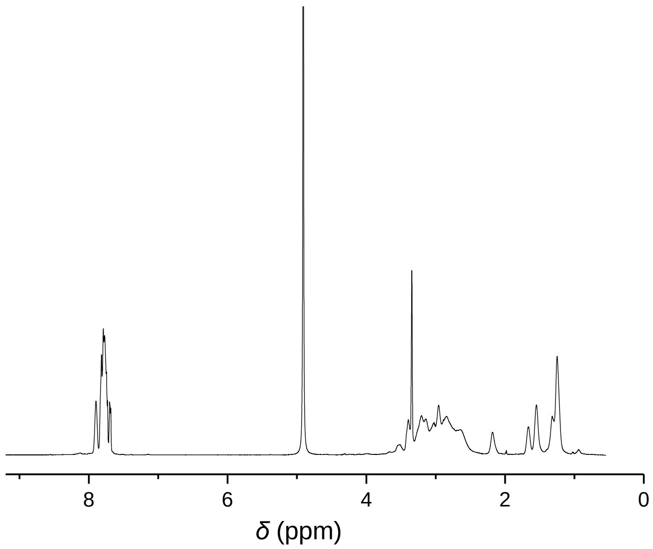

Figure S8. ^{1}H-NMR (500 MHz, MeOD-d_4) spectrum of P-TPP.

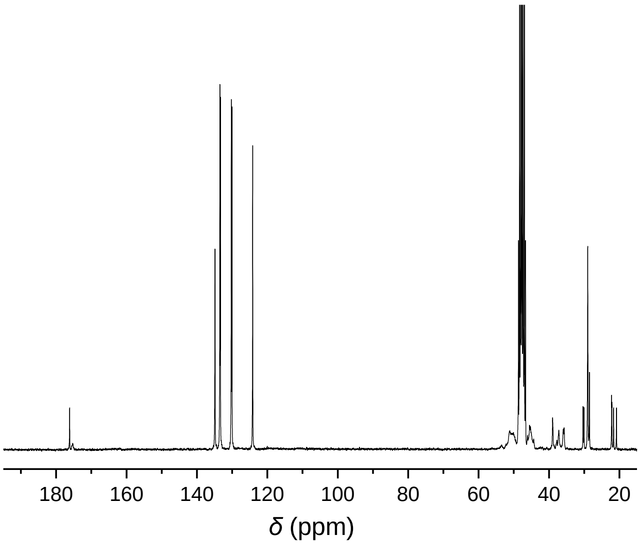

Figure S9. ^{13}C-NMR (125.1 MHz, MeOD-d_4) spectrum of P-TPP.

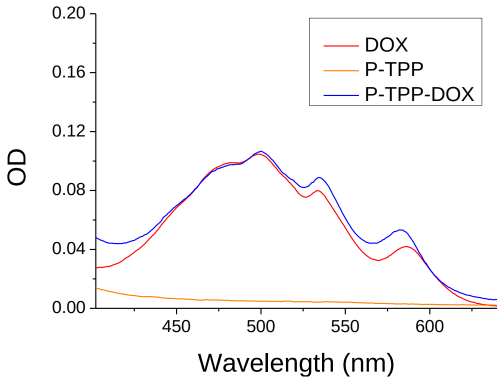

Figure S10. UV-Vis spectra of the carrier P-TPP, free DOX and P-TPP-DOX (DOX concentration of 9.3 μM) in ethanol.

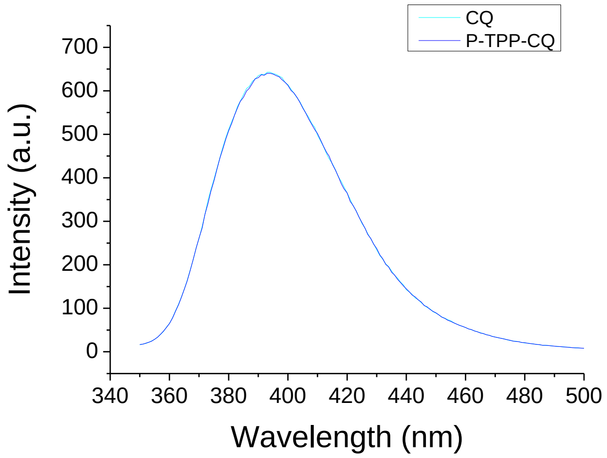

Figure S11. Fluorescence spectra of CQ or P-TPP-CQ (CQ concentration of 3.5 μM) in ethanol.
